# An “on-off-on” fluorescence probe for glyphosate detection based on Cu^2+^ modulated g-C_3_N_4_ nanosheets

**DOI:** 10.3389/fchem.2022.1036683

**Published:** 2022-09-30

**Authors:** Yingfeng Qin, Ruiqi Huang, Gao-Jie Ye

**Affiliations:** Guangxi Key Laboratory of Bioactive Molecules Research and Evaluation, Key Laboratory of Biological Molecular Medicine Research (Guangxi Medical University), Education Department of Guangxi Zhuang Autonomous Region, Pharmaceutical College and School of Basic Medical Sciences, Guangxi Medical University, Nanning, China

**Keywords:** glyphosate, G-C3N4 nanosheets, Cu^2+^, fluorescence, on-off-on

## Abstract

The analysis of glyphosate is essential to agricultural production, environment protection and public health. Herein, we proposed a fast and convenient “on-off-on” fluorescence platform for sensitive detection of glyphosate *via* Cu^2+^ modulated g-C_3_N_4_ nanosheets. The fluorescence of the system was quenched by Cu^2+^. With the presence of glyphosate, the fluorescence could be restored due to the formation of Cu^2+^- glyphosate complex. The proposed method was cost-effective with label-free and enzyme-free. Moreover, it exhibits high sensitivity with a low detection limit of 0.01 μg/ml. Furthermore, the proposed method has been successfully monitored glyphosate in real samples.

## 1 Highlights

• A fast and convenient “on-off-on” fluorescence platform based on Cu^2+^ modulated g-C3N4 nanosheets for sensitive detection of glyphosate was developed.
• This method is cost-effective and does not need any labeling, enzyme or other complex processes.
• The proposed method exhibits high sensitivity and has a good analysis performance in complex samples.

## 2 Introduction

The use of pesticides is highly merited to improve crop yields and products quality. Among many pesticides, glyphosate has become one of the most widely applied herbicides because of its high efficiency, broad-spectrum, non-selective, and low toxicity (Valle et al., 2019). Nevertheless, the misuse of glyphosate can cause its high residues in soil, water, and food, and then produce some problems regarding environmental pollution and health hazards. Moreover, glyphosate exposure has adverse effects on the endocrine system, central nervous system and cell cycle (Gasnier et al., 2009; Wozniak et al., 2020). Besides that, glyphosate has been listed as a potential carcinogen (Guyton et al., 2015). Therefore, constructing a facile, low-cost, and high-efficiency method for glyphosate detection is of great importance for public health and environmental protection.

Currently, some traditional approaches have been applied to analyze glyphosate such as capillary electrophoresis (Muñoz et al., 2019), gas chromatography (Royer et al., 2000; Ding et al., 2015), high-performance liquid chromatography (Sun et al., 2017; Surapong and Burakham, 2021), Chromatography-mass spectrometry (Schütze et al., 2021; Pérez-Mayán et al., 2022), and enzyme-linked immunosorbent assay (González-Martínez et al., 2005). However, these methods tend to require sophisticated instrumentation, tedious pretreatments, long testing times or tedious operation. To overcome such limitations, several techniques have been proposed (Xu et al., 2018; Qin et al., 2020; Ding et al., 2021; Wu et al., 2022; Zhao et al., 2022). Among them, fluorescence methods have been receiving great attention owing to their superior analytical performances such as simplistic, rapid, and sensitive. Especially, fluorescent probes can be applied to develop label-free fluorescent platforms for target analysis (Liu et al., 2020; Zhang et al., 2020; Liu et al., 2021).

As a promising kind of 2D nanomaterials, g-C3N4 nanosheets (CN NNS) have attracted much attention because of their low cost, easy synthesis, excellent catalytic performance, metal-free, water solubility and excellent biocompatibility (Dong et al., 2016). Up to now, CN NNS have emerged in biosensor, imaging and photocatalysis (Guo et al., 2011; Salehnia et al., 2017; Zhang et al., 2021; Zheng et al., 2021). Intriguingly, CN NNS not only have excellent fluorescence properties, but also the fluorescence can be quenched by some metal ions including Fe^3+^, Hg^2+^, Eu^3+^, and Cr^6+^(Rong et al., 2015a; Zhuang et al., 2017; Ti et al., 2021; Wang et al., 2021). Recently, Chen group reported a label-free fluorescence sensor for detection of Fe^3+^ and ascorbic acid *via* CN NNS (Guo et al., 2018). Duan group used CN NNS to establish a facile fluorescence approach for 6-Thioguanine and Hg^2+^ (Duan et al., 2018). This property can be used to develop novel sensor strategies for metal ion detection or some other targets detection which were mediated by these metal ions.

In the present work, a facile and effective “on-off-on” fluorescence sensor based on CN NNS was developed for glyphosate. The CN NNS were prepared by one-step process. The fluorescence of the CN NNS could be quenched by Cu^2+^. Then, the fluorescence gradually increased with the addition of glyphosate due to Cu^2+^ preferentially coordinated with glyphosate. The quantitation of glyphosate could be achieved according to the change of fluorescence. Thus, the proposed approach not only provided a novel sensor platform for glyphosate but also exhibited a potential application in environmental safety and biological fields.

## 3 Materials and methods

### 3.1 Reagents and materials

Glyphosate, dicyanamide, copper chloride (CuCl_2_), and other pesticides (such as carbendazim, carbaryl, parathion, malathion, chlorpyrifos, diazinon, omethoate) were gained from Aladdin Reagent Co., Ltd. (Shanghai, China). Tris was purchased from Solarbio Science and Technology Co., Ltd. (Beijing, China). Ultrapure water was gained from a Milli-Q Integral 15 system (Millipore) and used throughout the work. Water samples were obtained from Yongjiang river in Nanning City and the lake water of Guangxi Medical University campus.

### 3.2 Preparation of CN NNS nanosheets

The CN NNS were synthesized according to the previously reported literature (Rong et al., 2015b). Briefly, the alumina crucible containing 10 g of ground dicyandiamide was placed in a muffle furnace, heated to 550°C at a heating rate of 3°C/min, reacted for 2 h, and then cooled to 25°C at the same rate. As a result, the bulk CN NNS was acquired. After that, 1 g of bulk CN NNS was put into 100 mL10 M HNO_3_ and refluxed for 16 h at 25°C. Then, the refluxed product was collected through centrifugation at 10000 rpm and washed to neutrality with water. The obtained precipitate was dispersed in 50 ml water for 6 h through a 2D nanomaterial stripper. Finally, the CN NNS solution was stored at 4°C.

### 3.3 Analysis of glyphosate

First, the stock solution of glyphosate was diluted with water to different concentrations. Then, 2 μL of 0.2 mM Cu^2+^, 10 μL of different concentrations of glyphosate, 5 μL CN NNS and 183 μL 20 mM Tris-HCl buffer (pH 6.0) reacted at room temperature for 10 min with a total volume of 200 μL. Subsequently, the fluorescence spectra of the samples were collected using an FL-8500 fluorescence spectrometer (PerkinElmer, United States) with the excitation wavelength at 308 nm.

### 3.4 Determination of glyphosate in real samples

First, the samples of river water and lake water were filtered with 0.22 μm membrane to remove solid impurities, respectively. After that, the analysis of glyphosate in the real water samples were carried out as described above processing procedure (2.3 Analysis of glyphosate).

## 4 Results and discussion

### 4.1 Characterization

**FIGURE 1:**
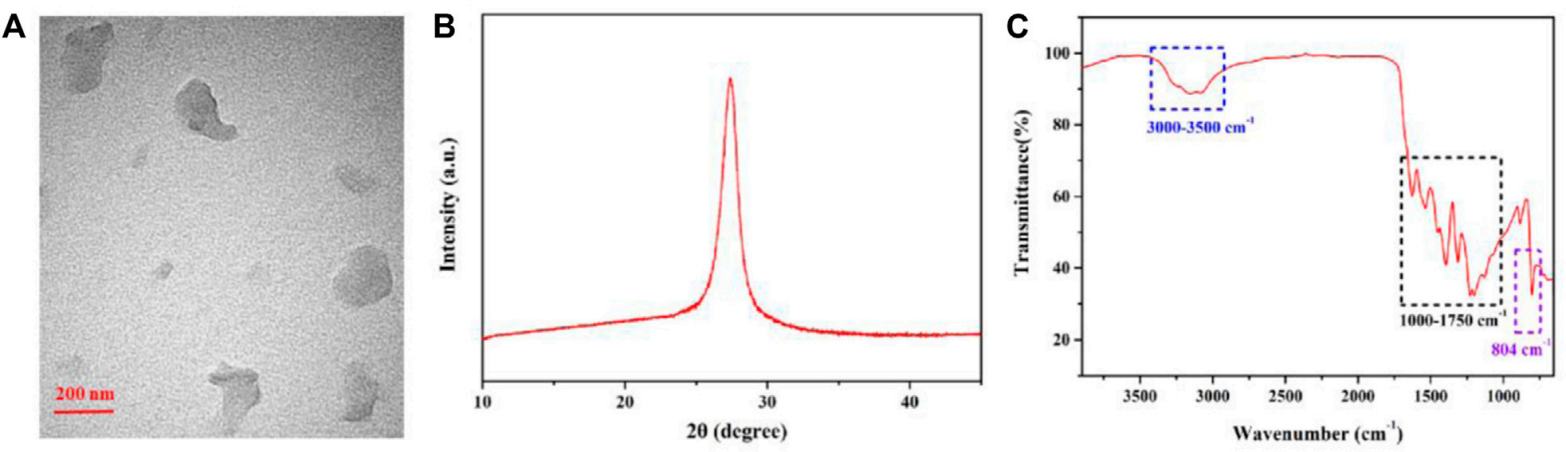
The Characterization of CN NNS **(A)** TEM image; **(B)** XRD image; **(C)** FT-IR spectrum.

The morphology of the obtained CN NNS was characterized through transmission electron microscopy (TEM). Figure 1A exhibited a lamellar structure and a well-dispersed state by TEM image. The XRD image displayed a broad diffraction peak (002) at 27.6° in Figure 1B, which was consistent with previous reports (Sun et al., 2020). As shown in Figure 1C, FT-IR spectrum of the obtained CN NNS was analyzed. The broad bands peaked at 3,000 to 3,500 cm^−1^ were ascribed to N-H stretching. The bands peaked at 1,000 to 1750 cm^−1^ were attributed to C=N stretching and C=O stretching. A characteristic peak of appeared at 804 cm^−1^, which was due to the vibration of the triazine ring. The FT-IR results indicated the presence of carboxyl, amino and hydroxy groups.

### 4.2 Optical properties of CN NNS nanosheets

**FIGURE 2:**
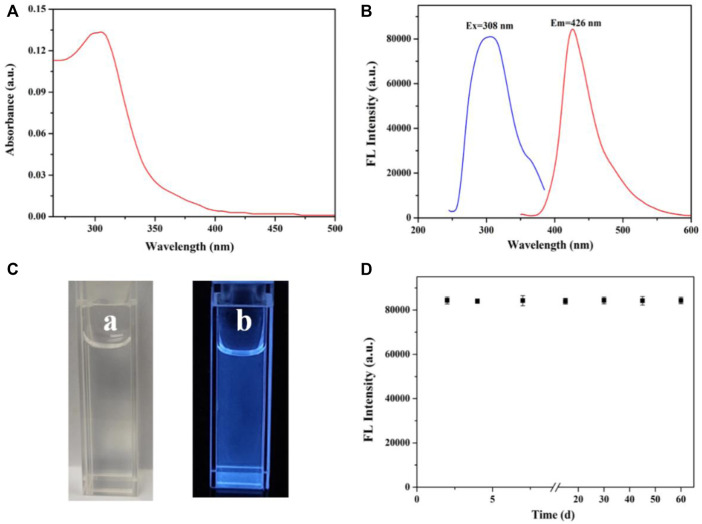
**(A)** UV-Vis spectra of CN NNS; **(B)** The fluorescence excitation and emission spectra of CN NNS; **(C)** The photos of the dispersion under room light **(A)** and 365 nm UV light **(B)**; **(D)**The fluorescence photostability of the dispersion.

In this work, the UV-Vis absorption spectroscopy and fluorescence spectra are used to reveal the optical properties of CN NNS. The UV-Vis absorption possessed a characteristic absorption peak at 308 nm in Figure 2A. In addition, Figure 2B shows the solution emitted a strong fluorescence emission at 426 nm with the excitation wavelength at 308 nm. Compared with daylight, brilliant blue fluorescence of the solution was clearly observed under the irradiation of 365 nm UV light (Figure 2C). Meanwhile, the fluorescence stability of the CN NNS dispersion was also researched. As displayed in Figure 2D, there is no obvious change in the intensity after the solution was stored for more than 2 months, implying outstanding stability of the dispersion.

### 4.3 Principle of the proposed sensor

**FIGURE 3:**
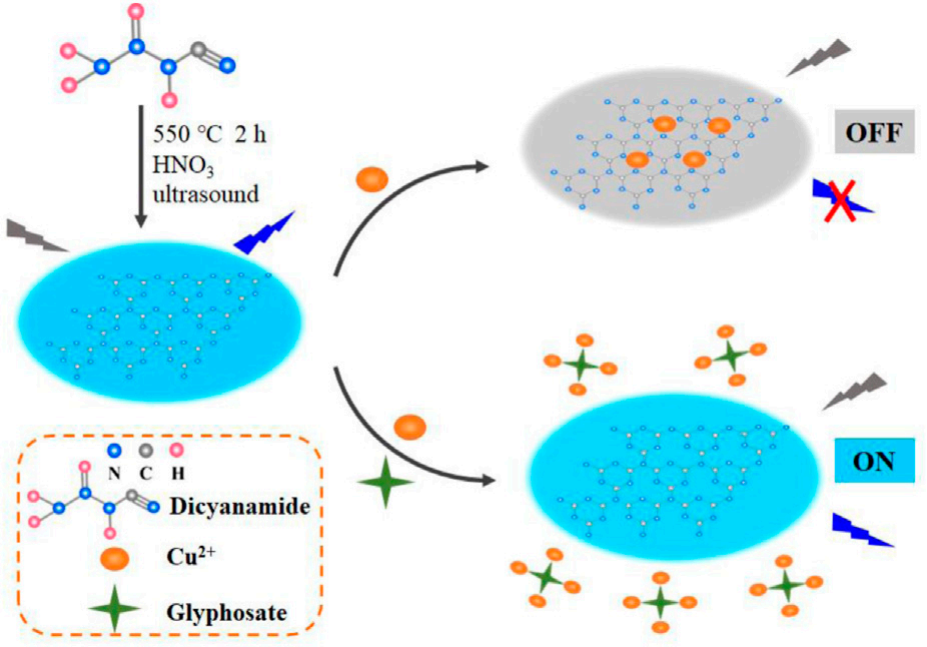
Schematic illustration of fluorescence sensor for glyphosate detection *via* CN NNS.

The principle of this novel enzyme-free fluorescence sensor for glyphosate assay based on CN NNS is illustrated in Figure 3. Firstly, the blue CN NNS were prepared by simple synthesis using dicyanamide. In the absence of glyphosate, the fluorescence of system was greatly quenched by Cu^2+^, lending to a low fluorescence signal. In contrast, upon the addition of glyphosate, Cu^2+^ preferentially coordinated with glyphosate to form glyphosate-Cu^2+^complex due to the stronger interaction than CN NNS-Cu^2+^. As a result, a high fluorescence signal was obtained. Thus, the concentrations of glyphosate could be detected by the fluorescence change.

### 4.4 Feasibility of the sensor

**FIGURE 4:**
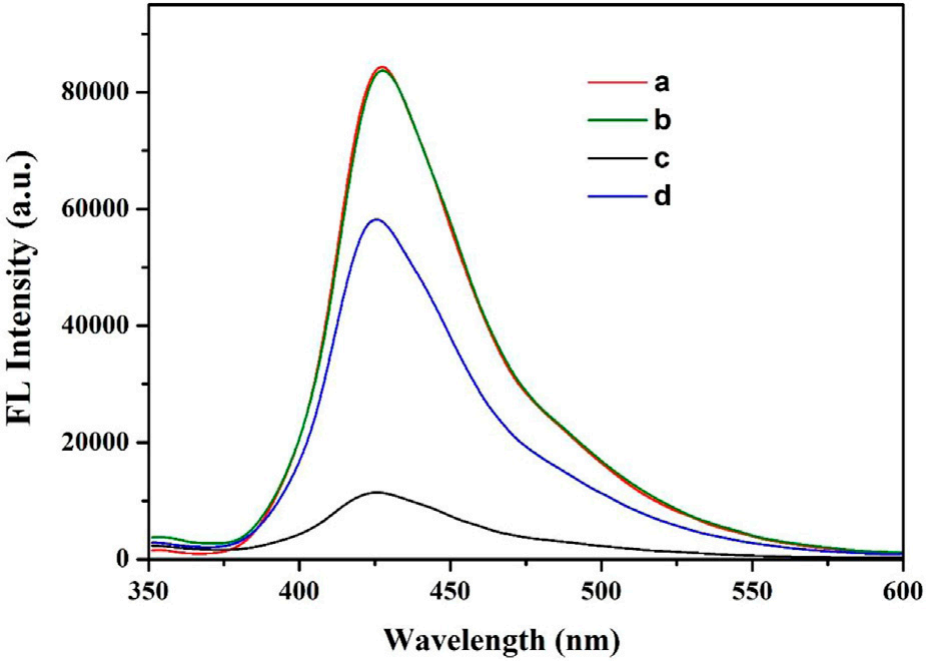
Fluorescence emission spectra of different sample solutions. Sample a: CN NNS; Sample b: CN NNS + glyphosate; Sample c: CN NNS + Cu^2+^; Sample d: CN NNS +Cu^2+^+ glyphosate. The concentrations of CN NNS, Cu^2+^ and glyphosate were 5 μg/mL, 2 μM and 5 μg/mL, respectively.

In order to verify the feasibility of this sensor strategy, the fluorescence emission spectra of the reaction solutions were detected. Figure 4 showed that the CN NNS had very high current fluorescence (curve a). After the introduction of glyphosate, no obvious change in fluorescence intensity at 426 nm was observed in curve a and curve b, manifesting that glyphosate had no effect on CN NNS. However, when Cu^2+^ was added to the system, the fluorescence intensity decreased significantly (curve c) due to the formation of the Cu^2+^-CN NNS complex. After glyphosate and Cu^2+^ were added, we observed that the fluorescence intensity of system increased significantly (curve d) because glyphosate exhibits a stronger combination ability with Cu^2+^. Above results illustrated that the fluorescence method using CN NNS and Cu^2+^ was feasible for glyphosate detection.

### 4.5 Optimization of assay conditions

For achieving the best assay performances, several main factors including the concentration of Cu^2+^, pH of reaction system, and the reaction time were optimized.

**FIGURE 5:**
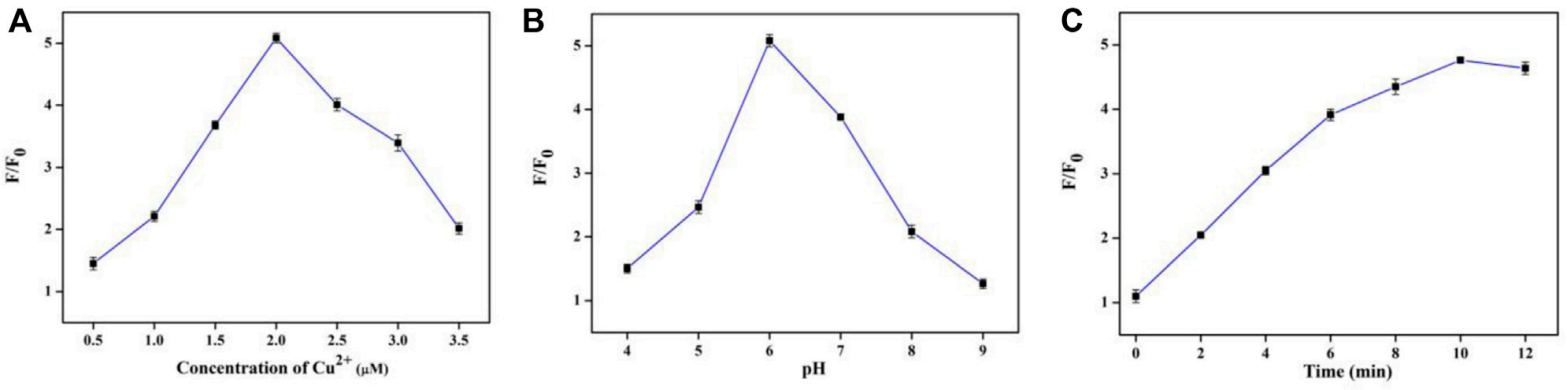
Optimization of assay conditions. The effect of Cu^2+^ concentration **(A)**, pH value **(B)**, and incubation time **(C)**.

The concentration of Cu^2+^ was first investigated. As seen in Figure 5A, the value of F/F_0_ increases with Cu^2+^ concentration from 0.5 μM to 2 μM. The maximum F/F_0_ value was obtained at 2 μM. Afterwards, the value of F/F_0_ decreased gradually. Thus, 2 μM was the optimal concentrations of Cu^2+^. In addition, the pH of reaction system was also optimized. The value of F/F_0_ increased on increasing pH from 4.0 to 6.0, and the F/F_0_ value reached a maximum when the pH was 6.0. However, after the pH exceeds 6.0, the F/F_0_ value gradually decreases as the pH value increases (Figure 5B). Thus, 6.0 was used as the optimum pH of the reaction system. Finally, effect of incubation time was also examined. The value of F/F_0_ increased with the increase reaction time and sustained a stable value at 10 min (Figure 5C). So, the optimal incubation time was 10 min.

### 4.6 Fluorescence assay for glyphosate

**FIGURE 6:**
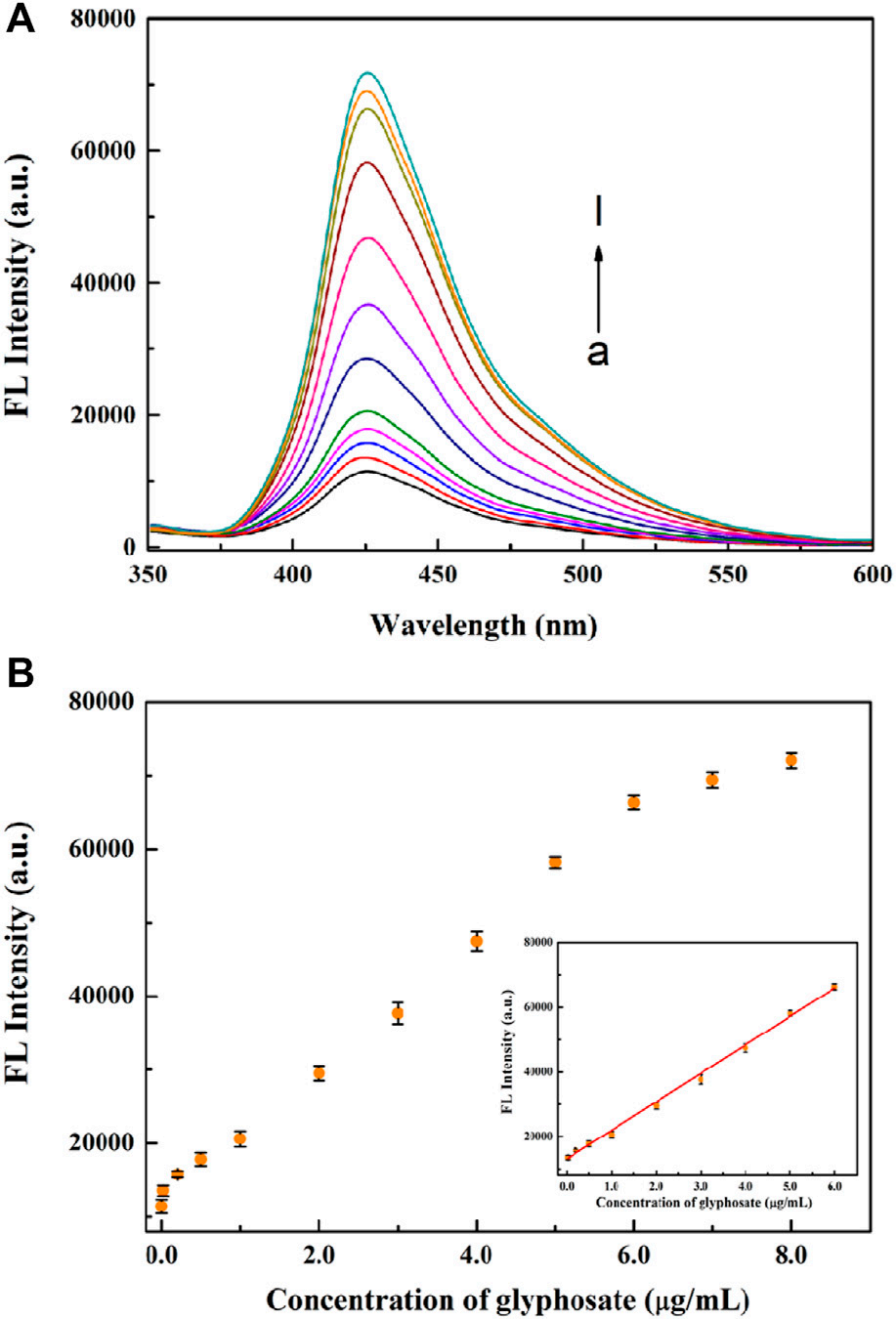
**(A)** The response of fluorescence emission spectra with different concentrations of glyphosate. The concentrations of glyphosate from a to l were 0, 0.02, 0.2, 0.5, 1, 2, 3, 4, 5, 6, 7 and 8 μg/ml, respectively. **(B)** The relationship between the fluorescence intensity and glyphosate concentrations. Inset: linear curve for glyphosate (0.02–6 μg/ml).

Based on the optimal conditions, various concentrations of glyphosate were analyzed. As illustrated in Figure 6A, the fluorescence intensity at 426 nm increases as the concentration of glyphosate increased from 0 to 8.0 μg/ml. The fluorescence intensity and the glyphosate concentration in the ranged from 0.02 μg/ml to 6.0 μg/ml shows a good linear relationship (inset of Figure 6B). The linear equation was F = 8,814.14C + 13143.35 (C: the concentration of glyphosate, F: the fluorescence intensity at 426 nm, R^2^ = 09968). The limit of detection is 0.01 μg/ml, which is substantially lower than the maximum residue of 0.7 μg/ml in drinking water by the US EPA and GB5749-2022 (Drinking Water Standards and Health Advisories., 2018; Standardization Administration of China., 2022). The sensitivity is comparable to or better than most other methods for glyphosate assay (Chang et al., 2016; Hou et al., 2020; Tai et al., 2022).

### 4.7 Selectivity study

**FIGURE 7:**
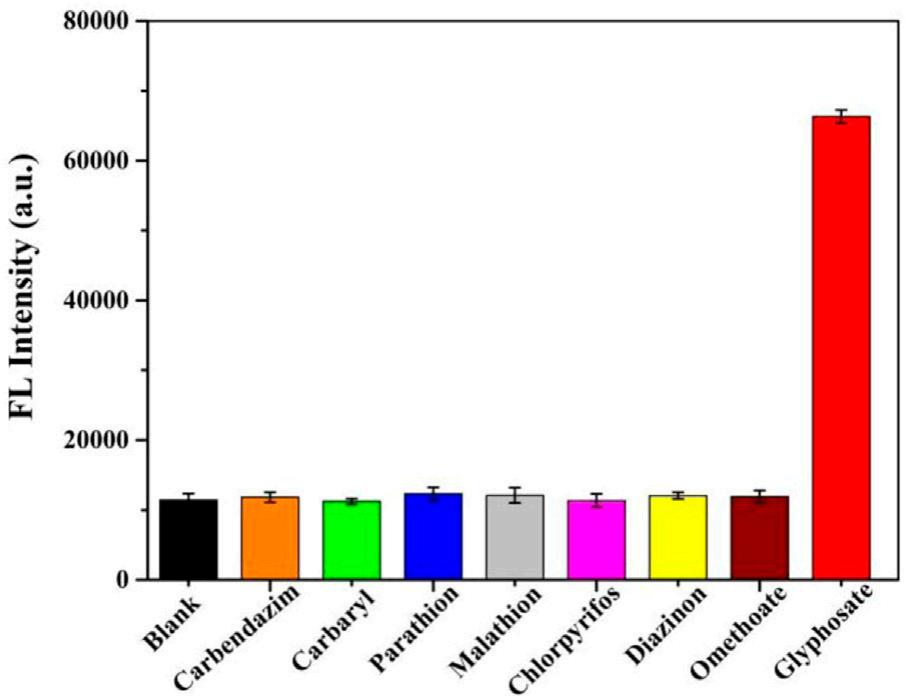
Selectivity of the present method for glyphosate. The concentration was 5 μg/ml for glyphosate, and the concentrations were 100 μg/ml for other pesticides.

The selectivity of this sensor was verified by detecting other pesticides including carbendazim, carbaryl, parathion, malathion, chlorpyrifos, diazinon and omethoate. As displayed in Figure 7, compared to other pesticides, only glyphosate could induce a remarkable fluorescence enhancement. These results indicated that the present assay has good selectivity for glyphosate detection.

### 4.8 Analysis of real samples

To evaluate the applicability of the present method in real samples, the recovery experiments were estimated in river water and lake water samples. The samples were spiked with different concentrations of glyphosate and detected. As can be seen in Table 1, The recoveries ranged from 96.5 to 104.4%. These results indicated the proposed method had great potential for glyphosate detection in complicated real samples.

**TABLE 1:** Detection of glyphosate in river water and lake water samples.

Samples	Added (μg/mL)	Found (μg/mL)	Recovery (%)	RSD (%, *n* = 3)
river water				
1	0.200	0.205	102.7	2.1
2	1.000	0.972	97.2	3.5
3	5.000	5.218	104.4	2.9
lake water				
1	0.200	0.193	96.5	2.6
2	1.000	1.026	102.6	2.2
3	5.000	5.162	103.2	3.3

## 5 Conclusion

In summary, we constructed a novel “on-off-on” sensor for sensitive detection of glyphosate *via* CN NNS as fluorescence probe. The fluorescence change of system can be obtained by the combination between copper ions and nanosheets or glyphosate, achieving the detection of glyphosate. The developed method was convenient, low-cost and rapid without tedious procedures. It exhibits a high sensitivity with a detection limit of 0.01 μg/ml. Besides that, a satisfactory performance in actual samples was also obtained. Therefore, the developed approach is expected to possess potential ;application in environmental safety and biological fields.

## Data Availability

The original contributions presented in the study are included in the article/supplementary material, further inquiries can be directed to the corresponding author.
